# Complex multilevel and multivessel endovascular revascularization through an occluded femoral-popliteal bypass in a patient with chronic limb threatening ischemia

**DOI:** 10.1590/1677-5449.210057

**Published:** 2022-01-17

**Authors:** Marcel Voos Budal Arins, Antenor Alvarez

**Affiliations:** 1 Centro de Cardiologia Intervencionista y Terapéutica Endovascular Periférica, Hemodinamia Integral, Santiago del Estero, Argentina.

**Keywords:** chronic limb-threatening ischemia, endovascular, subintimal angioplasty, retrograde approach

## Abstract

Chronic limb-threatening ischemia (CLTI) represents the end stage of peripheral artery disease, a problem of growing prevalence and increased health care costs around the globe. CLTI is a highly morbid disease, incurring significant mortality, limb loss, pain, and diminished health-related quality of life. The major cause of non-traumatic lower extremity amputation are related to diabetes and CLTI. Between 2% to 3% of patients with peripheral artery disease present with a severe case of CLTI, a condition that is correlated with multilevel and multivessel arterial disease, calcification, and chronic total occlusions. Multiple technical strategies to successfully cross long occlusions in arterial segments have been described. Recanalization can be performed using endoluminal, subintimal, and retrograde techniques. We report a case of complex multilevel and multivessel endovascular revascularization through an occluded femoro-popliteal bypass in a patient with CLTI.

## INTRODUCTION

Chronic limb-threatening ischemia (CLTI) represents the end stage of peripheral artery disease (PAD), a problem of growing prevalence and increased health care costs around the globe. CLTI is a highly morbid disease that generates amputations, pain, and diminished health-related quality of life.^1^

The major cause of non-traumatic amputations is related to diabetes mellitus and CLTI. From 2 to 3% of patients with PAD present with a severe case of CLTI, a condition that is correlated with multilevel multivessel arterial disease, calcification, and chronic total occlusions.^2^

The rapid evolution in imaging scans and devices provides new opportunities to improve treatment and management with endoluminal, subintimal, or retrograde techniques in these patients with high risk of amputation.^3^

The protocol was approved by the institutional Ethics Committee (Hemodinamia Integral).

### Part I – Clinical situation

The patient was a 78-year old woman with a history of arterial hypertension, diabetes mellitus, dyslipidemia, former heavy smoking, chronic obstructive pulmonary disease (COPD), and femoral popliteal bypass in the right lower limb placed 7 years before due to severe intermittent claudication (Rutherford 3).

**Figure 1:**
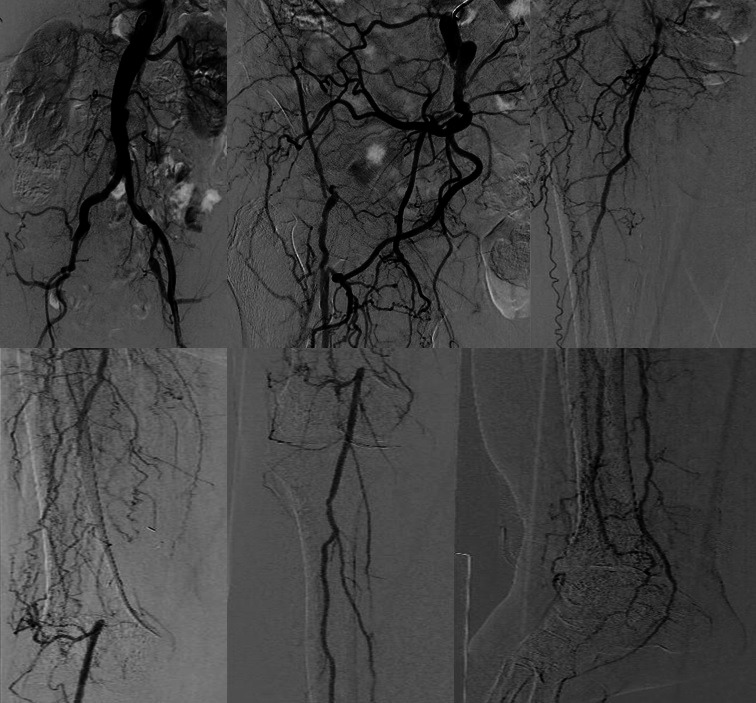
Image showing extensive multilevel and multivessel artery disease, with presence of calcification and chronic total occlusions.

The patient sought medical assistance due to CLTI (Rutherford 5), with pain at rest and an in the right heel ulcer (Wound, Ischemia and foot Infection [WIfI] classification 231). Right lower limb angiography revealed occlusion of external iliac, common femoral, superficial femoral, and anterior tibial arteries, occlusion of the P1 segment of the popliteal artery, and occlusion of the femoral popliteal bypass. The tibioperoneal trunk, the peroneal artery, and the posterior tibial artery were patent (Figure 1).

Open surgery was ruled out, due to patient's history of COPD.

### Part II – What was done

A puncture was made on the left common femoral artery, and a 45-cm 6 Fr Destination guiding sheath was placed into the right primitive iliac artery using the up-and-over technique.

There was a failed intent of anterograde revascularization with a 4 Fr vertebral catheter and 0.018” (V-18) and 0.035” hydrophilic guidewires.

**Figure 2:**
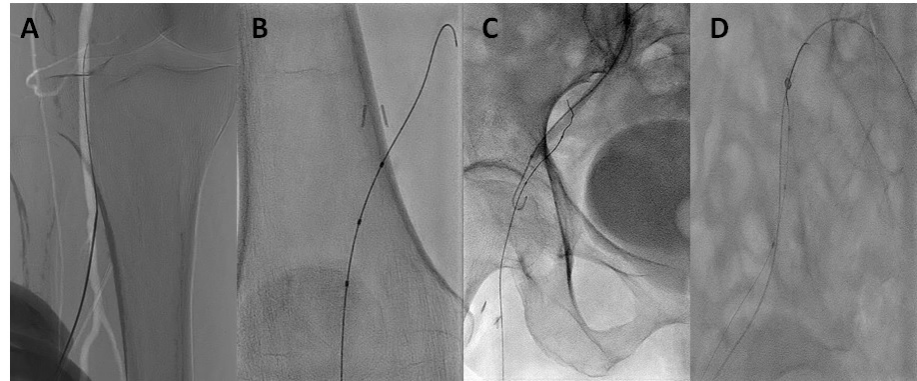
(A) Roadmap-guided puncture of the P3 segment of the popliteal artery; (B) Rubicon 18 support catheter and V-18 wire with a sheathless technique by inserting the femoral popliteal bypass using a retrograde approach; (C) CART technique; (D) Rendez-vous technique.

An approach was performed to the anterolateral surface of the right leg, and roadmap-guided puncture of the P3 segment of the popliteal artery was made using a 21G needle (Figure 2A).

With a Rubicon 18 support catheter and V-18 guidewire using a sheathless technique, the wire was advanced retrogradely until the external iliac artery, through the femoral popliteal bypass (Figure 2B). The guidewire was in a subintimal position, and it was not possible to reenter it via a retrograde path. Then, via an anterograde pathway, the CART technique was performed with a 5.0-mm balloon (Figure 2C), as well as the rendez-vous technique (Figure 2D). Finally, the retrograde guidewire was externalized through the left femoral access.

Pre-dilation of the popliteal artery was performed with a 6.0 mm × 100 mm balloon, and the common femoral artery and the right external iliac artery were pre-dilated with a 7.0 mm × 150 mm balloon.

A self-expandable Supera nitinol 5.5 mm × 200 mm stent was inserted into the popliteal artery and into the distal and medial portions of the bypass and a self-expandable Supera nitinol 6.5 mm × 180 mm stent was inserted into the proximal portion of the bypass and in the common femoral artery. Similarly, a self-expandable Epic nitinol 8.0 mm × 120 mm stent was placed in the external iliac artery and in the primitive iliac artery. Finally, endo-hemostasis of the retrograde puncture site was achieved with a 4.0 mm balloon.

**Figure 3:**
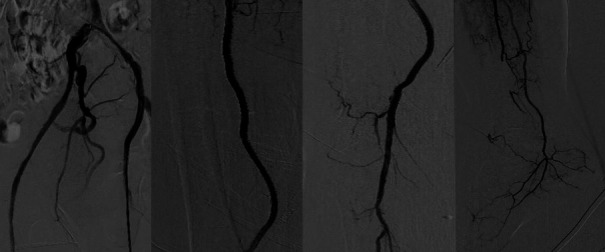
Final angiographic control follow-up showing good direct flow to the foot.

Angiographic control revealed good outcomes in the treated segments, embolization at the level of the distal peroneal artery, and good anterograde flow in the posterior tibial artery. Good flow was also observed in medial calcaneal branch of the posterior tibial artery that irrigates the angiosome corresponding to the ulcer site, as well as in both plantar arteries (Figure 3).

**Figure 4:**
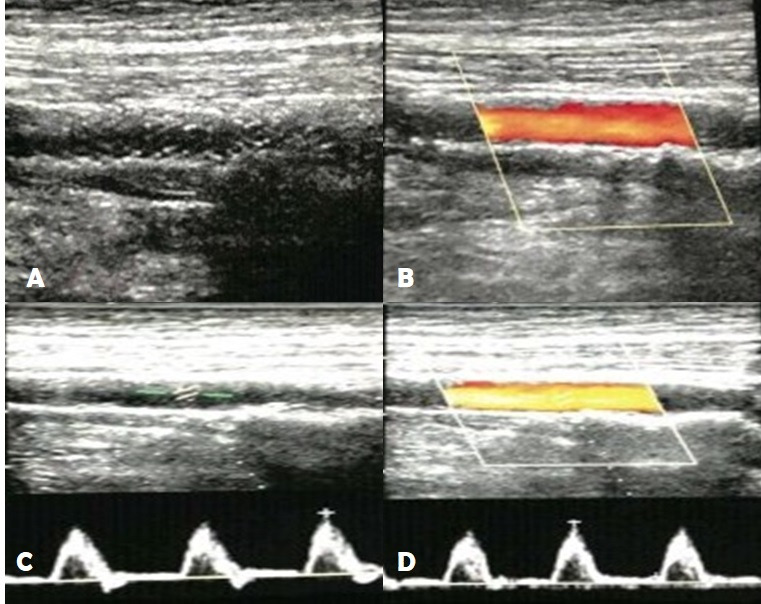
90-day post-angioplasty Doppler echocardiograph. (A) B mode: Stent in the popliteal artery with no signs of fracture; (B) Doppler: Stent with laminar flow, with no turbulences or signs of neointimal hyperplasia; (C) B mode: Distal popliteal artery with no significant atherosclerotic plaques; (D) Doppler of distal popliteal artery: Three-phase curve with peak systolic velocity of 74 cm/s.

The patient underwent outpatient follow-up with physical examination and control Doppler echocardiography at 30, 60, and 90 days after angioplasty. Physical examination showed complete healing of heel ulcer. Control Doppler echocardiography revealed no signs of stent fracture, showing laminar flow, with no turbulences or signs of neointimal hyperplasia (Figure 4).

Informed consent is sufficient for the elaboration of the manuscript.

## DISCUSSION

CLTI is the result of the multilevel occlusive artery disease.^1^ Patients with CLTI have multiple comorbidities, and the selection of the initial treatment requires a personalized therapeutic focus that balances patient's clinical status and technical and anatomical limitations.^4^

The minimally invasive nature of endovascular therapy allows for it to be applied to most of the population, especially to those with a high number of comorbidities,^4^ like our patient.

During endovascular treatment of CLTI, it is essential to adapt the technical strategy of the procedure to clinical indications and to amputation risk.^5^

Since open surgery was ruled out, due to patient's high number of comorbidities, with high risk of amputation and a challenging and complex vascular anatomy, an aggressive endovascular strategy was chosen, including anterograde accesses and endoluminal, subintimal, reentry (CART) techniques, as a technique of reconnection with the anterograde approach (rendez-vous).

These techniques, associated with good imaging quality and use of specific devices, provide good clinical outcomes in terms of limb salvage and amputation-free survival.

In conclusion, the decision of using complex endovascular techniques should be based on patient's clinical status and not only on vascular anatomy.

There are many techniques to approach complex anatomies. When they are appropriately combined, following clinical needs, it is possible to achieve good outcomes, preventing amputations and reducing patients' morbidity and mortality.
