# Supplementary material for: Complex multilevel and multivessel endovascular revascularization through an occluded femoral-popliteal bypass in a patient with chronic limb threatening ischemia
Source: J Vasc Bras. 2022 Jan 17;21:e20210057. doi: 10.1590/1677-5449.210057 (PMC8786082; doi:10.1590/1677-5449.210057)
Supplement: Translation

# Translation: Revascularización endovascular compleja multinivel y multivaso a través de un *bypass* fémoro-poplíteo ocluído en paciente con isquemia crónica con amenaza para las extremidades inferiores

**Authors:** Marcel Voos Budal Arins, Antenor Alvarez

**Affiliations:** 1 Centro de Cardiologia Intervencionista y Terapéutica Endovascular Periférica, Hemodinamia Integral, Santiago del Estero, Argentina.

**Keywords:** isquemia crónica con amenaza para las extremidades inferiores, endovascular, angioplastia subintimal, acceso retrógrado

## Abstract

La isquemia crónica con amenaza para las extremidades inferiores (ICAEI) representa el estadio final de la enfermedad arterial periférica, un problema de prevalencia creciente que conlleva el aumento de los costos de salud en todo el mundo. La ICAEI es una enfermedad con elevada morbilidad, generando mortalidad significativa, pérdida de miembros, dolor y disminución de la calidad de vida. La principal causa de amputaciones no-traumáticas de miembros inferiores está relacionada a la diabetes y a la ICAEI. Entre un 2% y 3% de los pacientes con enfermedad arterial periférica se presentan con un caso grave de ICAEI, condición que se correlaciona con enfermedad arterial multinivel y multiarterial, calcificación y oclusiones totales crónicas. Se describieron varias estrategias técnicas para cruzar con éxito largas oclusiones en segmentos arteriales. Se puede realizar la recanalización utilizando técnicas endoluminales, subintimales y retrógradas. Relatamos un caso de revascularización endovascular compleja multinivel y multiarterial a través de un *bypass* fémoro-poplíteo en una paciente con ICAEI.

## INTRODUCCIÓN

La isquemia crónica con amenaza para las extremidades inferiores (ICAEI) representa el estadio final de la enfermedad arterial periférica (EAP), un problema de prevalencia creciente que conlleva grandes costos de atención médica en todo el mundo. La ICAEI es una enfermedad con elevada morbimortalidad, generando amputaciones, dolor y disminución de la calidad de vida^1^.

La principal causa de amputaciones no traumáticas está relacionada a la diabetes *mellitus* y a la ICAEI. Entre un 2 a 3% de los pacientes con EAP se presentan con un caso grave de ICAEI, condición que se correlaciona con la enfermedad arterial multinivel y multivaso, calcificación y oclusiones totales crónicas^2^.

La rápida evolución tecnológica en imágenes y dispositivos ofrece nuevas oportunidades para mejorar el tratamiento y el abordaje con técnicas endoluminales, subintimales o por vía retrógrada en estos pacientes con alto riesgo de amputación^3^.

El protocolo fue aprobado por el Comité de Ética de la institución (Hemodinamia Integral).

### Parte I – Situación clínica

Se trata de una paciente femenina de 78 años de edad con antecedentes de hipertensión arterial, diabetes mellitus, dislipemia, extabaquismo severo, enfermedad pulmonar obstructiva crónica (EPOC) y *bypass* fémoro-poplíteo en miembro inferior derecho hace 7 años por claudicación intermitente grave (Rutherford 3).

La paciente consulta por ICAEI (Rutherford 5), con dolor de reposo y una úlcera en el talón derecho [classificación *Wound, Ischemia and foot Infection* (WIfI) 231]. La angiografía del miembro inferior derecho reveló la oclusión de las arterias iliaca externa, femoral común, femoral superficial, tibial anterior, oclusión del segmento P1 de la arteria poplítea y oclusión del *bypass* fémoro-poplíteo. El tronco tibio-peroneo, la arteria peronea y la arteria tibial posterior se encontraban permeables (Figura 1).

**Figura 1:** Se observa extensa enfermedad arterial multinivel y multivaso, con presencia de calcificación y de oclusiones totales crónicas.

La paciente fue descartada para cirugía abierta por su antecedente de EPOC.

### Parte II – Qué se hizo

Se hizo punción de la arteria femoral común izquierda y colocación de introductor Destination 6 Fr de 45 cm en la arteria iliaca primitiva derecha por la técnica de *up and over*.

Hubo un intento fallido de revascularización anterógrada con un catéter de soporte vertebral 4 Fr y guías 0,018” (V-18) e hidrofílicas 0,035”.

Se realizó abordaje por la cara anterolateral de la pierna derecha y punción guiada por *roadmapping* del segmento P3 de la arteria poplítea con aguja de 21G (Figura 2A).

**Figura 2:** (A) Punción guiada por roadmapping del segmento P3 de la arteria poplítea; (B) Catéter Rubicon 18 y guía V-18 con técnica sin introductor (*sheathless*) ingresando al *bypass* fémoro-poplíteo por vía retrógrada; (C) Técnica CART; (D) Técnica *rendezvous*.

Con un catéter de soporte Rubicon 18 y una guía V-18 con técnica sin introductor (*sheathless*), se logró progresar por vía retrógrada la guía hasta la arteria iliaca externa, a través del *bypass* fémoro-poplíteo (Figura 2B). La guía se encontraba en posición subintimal y no fue posible reentrar por vía retrógrada. Luego, por vía anterógrada, se realizó técnica CART con un balón de 5,0 mm de diámetro (Figura 2C) y la técnica *rendezvous* (Figura 2D), externalizándose la guía retrógrada por el acceso femoral izquierdo.

Se realizó pre-dilatación de arteria poplítea con balón de 6,0 mm de diámetro por 100 mm de longitud y de arteria femoral común e iliaca externa derecha con balón de 7,0 mm de diámetro por 150 mm de longitud.

Se implantó un stent autoexpandible de nitinol Supera de 5,5 mm de diámetro por 200 mm de longitud en arteria poplítea y porciones distal y media del *bypass*. Además, hubo la implantación de de stent autoexpandible de nitinol Supera de 6,5 mm de diámetro por 180 mm de longitud en porción proximal del *bypass* y la arteria femoral común; y también un stent autoexpandible de nitinol Epic de 8,0 mm de diámetro por 120 mm de longitud en las arterias iliaca externa e iliaca primitiva. Por fin, se logró endohemostasia del sitio de punción retrógrada con un balón de 4,0 mm de diámetro.

El control angiográfico reveló buen resultado en los segmentos tratados, embolización a nivel de la porción distal de la arteria peroneal y buen flujo anterógrado en la arteria tibial posterior. Se observó buen flujo en la rama calcánea media de la arteria tibial posterior que irrigaba el angiosoma correspondiente al sitio de la úlcera y buen flujo en ambas arterias plantares (Figura 3).

**Figura 3:** Control angiográfico final con buen flujo directo hacia el pié.

La paciente fue seguida de forma ambulatoria con examen físico y control con ecografía Doppler a los 30, 60 y 90 días post-angioplastia. El examen físico mostró curación completa de la úlcera en el talón. Control con ecografía Doppler no reveló señales de fractura de los stents, demostrando flujo laminar, sin turbulencias ni señales de hiperplasia neointimal (Figura 4).

**Figura 4:** Ecografía Doppler a los 90 días post-angioplastia. (A) Modo B: Stent en arteria poplítea sin señales de fractura; (B) Doppler: Stent con flujo laminar, sin turbulencias ni señales de hiperplasia neointimal; (C) Modo B: Arteria poplítea distal sin placas aterosclerótica significativas; (D) Doppler arteria poplítea distal: Curva trifásica con velocidad de pico sistólica de 74 cm/s.

El consentimiento informado es suficiente para la confección del manuscrito.

## DISCUSIÓN

La ICAEI es el resultado de la enfermedad arterial oclusiva multinivel^1^. Los pacientes con ICAEI tienen múltiples comorbilidades, y la elección del tratamiento inicial requiere un enfoque terapéutico personalizado que equilibre la clínica del paciente con las limitaciones técnicas y anatómicas^4^.

La característica mínimamente invasiva de la terapéutica endovascular permite que sea aplicada a gran parte de la población, especialmente a los que tienen elevada comorbilidad^4^, como es el caso de nuestra paciente.

Durante el tratamiento endovascular de la ICAEI, es fundamental adaptar la estrategia técnica del procedimiento a las indicaciones clínicas y al riesgo de amputación^5^.

Por tratarse de una paciente descartada para cirugía abierta por elevada comorbilidad, con alto riesgo de amputación y con una anatomía vascular desafiante y compleja, se optó por una estrategia endovascular agresiva con accesos anterógrado, retrógrado, técnicas endoluminales, subintimales, de reentrada (CART) y de reconexión con el acceso anterógrado (rendezvous).

Estas técnicas, asociadas a la buena calidad de imagen y a la utilización de dispositivos específicos, proporcionan buenos resultados clínicos en términos de salvataje del miembro y supervivencia libre de amputación.

En conclusión, la decisión de utilizar técnicas endovasculares complejas debe estar guiada por el estado clínico del paciente y no sólo por la anatomía vascular.

Contamos con muchas técnicas para abordar anatomías complejas y, al combinarlas de forma adecuada, siguiendo las necesidades clínicas, podemos lograr buenos resultados, evitando amputaciones y disminuyendo la morbimortalidad de los pacientes.
